# Type I Saikosaponins A and D Inhibit Osteoclastogenesis in Bone Marrow-Derived Macrophages and Osteolytic Activity of Metastatic Breast Cancer Cells

**DOI:** 10.1155/2015/582437

**Published:** 2015-03-29

**Authors:** Ji-Eun Shin, Hyun-Jeong Kim, Ki-Rim Kim, Sun Kyoung Lee, Junhee Park, Hyungkeun Kim, Kwang-Kyun Park, Won-Yoon Chung

**Affiliations:** ^1^Department of Applied Life Science, The Graduate School, Yonsei University, 50 Yonsei-ro, Seodaemun-gu, Seoul 120-749, Republic of Korea; ^2^Department of Oral Biology, Oral Cancer Research Institute and BK21 PLUS Project, Yonsei University College of Dentistry, 50-1 Yonsei-ro, Seodaemun-gu, Seoul 120-752, Republic of Korea; ^3^Department of Dental Hygiene, Kyungpook National University, Sangju 742-711, Republic of Korea; ^4^Department of Dentistry, The Graduate School, Yonsei University, 50 Yonsei-ro, Seodaemun-gu, Seoul 120-749, Republic of Korea

## Abstract

Many osteopenic disorders, including a postmenopausal osteoporosis and lytic bone metastasis in breast and prostate cancers, are linked with a hyperosteoclast activity due to increased receptor activator of nuclear factor kappa-B ligand (RANKL) expression in osteoblastic/stromal cells. Therefore, inhibition of RANKL-induced osteoclastogenesis and osteoclast-induced bone resorption is an important approach in controlling pathophysiology of these skeletal diseases. We found that, of seven type I, II, and III saikosaponins isolated from *Bupleurum falcatum*, saikosaponins A and D, type I saikosaponins with an allyl oxide linkage between position 13 and 28 and two carbohydrate chains that are directly attached to the hydroxyl groups in position 3, exhibited the most potent inhibition on RANKL-induced osteoclast formation at noncytotoxic concentrations. The stereochemistry of the hydroxyl group at C16 did not affect their activity. Saikosaponins A and D inhibited the formation of resorptive pits by reducing the secreted levels of matrix metalloproteinase- (MMP-) 2, MMP-9, and cathepsin K in RANKL-induced osteoclasts. Additionally, saikosaponins A and D inhibited mRNA expression of parathyroid hormone-related protein as well as cell viability and invasion in metastatic human breast cancer cells. Thus, saikosaponins A and D can serve as a beneficial agent for the prevention and treatment of osteoporosis and cancer-induced bone loss.

## 1. Introduction

Bone is a specialized connective tissue composed of both mineral and organic phases that is exquisitely designed for its role as the load-bearing structure of a body. Unlike other durable structures, such as teeth, tendons, and cartilage, bone is continuously renewed by the process of bone remodeling in which pockets or trenches of bone are removed from the surfaces of the trabecular and cortical bone and subsequently replaced by new bone. The process of bone remodeling consists of both osteoclastic bone resorption and osteoblastic bone formation [1]. Under healthy physiological conditions, activities of osteoclasts and osteoblasts are tightly controlled by both systemic and local factors, maintaining bone mineral density and bone microarchitecture. However, an imbalance in osteoclasts and osteoblasts activities, mostly in favor of a hyperosteoclast activity, is responsible for pathophysiology of several skeletal diseases, including osteoporosis and metastatic bone diseases [1].

Current medical treatment of osteoporosis and breast cancer bone metastasis is anti-bone resorptive agents, including bisphosphonates and denosumab [2–4]. Bisphosphonates and denosumab, a receptor activator of nuclear factor kappa-B ligand (RANKL) inhibitor, delay time to first skeletal related event. However, these drugs do not prevent development of bone metastasis and also do not prolong survival [4]. Thus, emerging therapies, including cathepsin K, matrix metalloproteinase, or nonreceptor tyrosine kinase Src inhibitors, are being introduced for prevention and treatment of osteoporosis and cancer bone metastasis. In addition, many medicinal plants and their active components in traditional Chinese medicine have been proven to possess efficacy on osteoporosis and cancer and thereby have attracted attention as a source of new drugs with better potency and safety for prevention and long-term treatment [5, 6].

Radix bupleuri is an Asian traditional herbal medicine used either alone or in combination with other ingredients for treating influenza, fever, inflammatory disorders, infectious diseases, hepatitis, menstrual disorders, and cancer [7, 8].* Bupleurum* species (Umbelliferae) are formally listed in the Chinese and Japanese pharmacopoeias and in the WHO monographs of the commonly used 150 medicinal plants [9, 10]. Saikosaponins, the most abundant triterpene saponins found in* Bupleurum* species, have been known to exhibit a variety of pharmacological activities, including immunomodulatory, antioxidant, hepatoprotective, and estrogen-like activities [11–13]. Saikosaponins B_1_, B_2_, and D stimulated prostaglandin E_2_ release from peritoneal macrophages and C6 rat glioma cells; however, saikosaponins A and C were inhibitory [13–15]. Saikosaponin B_2_ induced apoptosis in B16 melanoma cells and saikosaponin C increased the angiogenic activity of endothelial cells [16, 17]. Saikosaponin A induced apoptosis in human breast and colon carcinoma cell lines [18, 19]. Saikosaponin D inhibited proliferation of human undifferentiated thyroid carcinoma cells [20] and enhanced radiosensitivity of hepatoma cells [21]. In addition, a recent study reported osteoclast-inhibiting activity of saikosaponins B_1_ and B_2_ [22].

**Figure 1:**
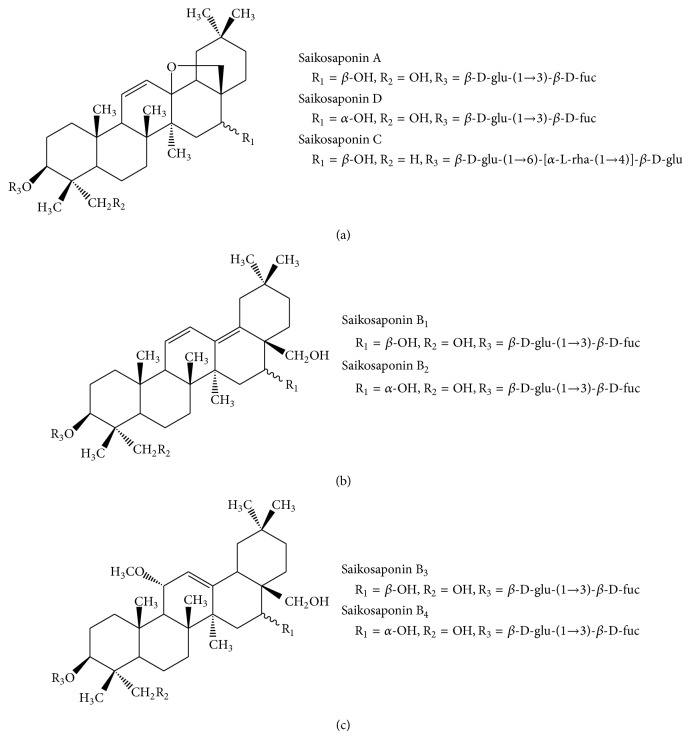
Chemical structure of saikosaponins. (a) Type I saikosaponins, (b) type II saikosaponins, and (c) type III saikosaponins.

In the present study to detect novel compounds with preventive and therapeutic activity on osteoclast-mediated bone diseases, we investigated the inhibitory activity of seven saikosaponins isolated from* B. falcatum* (Figure 1) on RANKL-induced osteoclastogenesis of murine bone marrow macrophages (BMMs). We further determined the effects of saikosaponins A and D on bone-resorbing proteases derived from osteoclasts and the resulting bone resorption. In addition, we showed their effect on the viability, invasion, and production of a key osteolytic factor, parathyroid hormone-related protein (PTHrP), in metastatic breast cancer cells.

## 2. Materials and Methods

### 2.1. Chemicals

Saikosaponins A, B_1_, B_2_, B_3_, B_4_, C, and D isolated from* B. falcatum* L. were provided by Professor Kim [23, 24]. The saikosaponins were dissolved in dimethyl sulfoxide (DMSO) and then diluted with culture medium just before use. Minimum essential medium alpha (*α*-MEM), Dulbecco's modified Eagle's medium (DMEM), fetal bovine serum (FBS), Dulbecco's phosphate buffered saline (PBS), antibiotic-antimycotic mixture, and 0.25% trypsin-EDTA were purchased from Gibco BRL (Grand Island, NY). Histopaque-1083, 3-(4,5-dimethylthiazol-2-yl)-2,5-diphenyltetrazolium bromide (MTT), and dimethyl sulfoxide (DMSO) were obtained from Sigma-Aldrich (St. Louis, MO). Recombinant mouse RANKL was purchased from Koma Biotech (Seoul, Republic of Korea) and recombinant mouse macrophage-colony stimulating factor (M-CSF) was obtained from R&D system (Minneapolis, MN). TGF-*β* was purchased from PeproTech (Rocky Hill, NJ). All reagents used in this study were of analytical grade.

### 2.2. Cell Culture

Mouse BMMs were isolated from the tibiae of 4-week-old male ICR mice by Histopaque density gradient centrifugation as described previously [25]. BMMs were cultured in *α*-MEM containing 10% FBS, 1% antibiotic-antimycotic mixture, and 30 ng/mL of M-CSF in a humidified atmosphere of 5% CO_2_ at 37°C. MDA-MB-231 human breast cancer cells (Korean Cell Line Bank, Seoul, Republic of Korea) were grown in DMEM containing 10% FBS and 1% antibiotic-antimycotic mixture in a humidified atmosphere of 5% CO_2_ at 37°C.

### 2.3. Cell Viability Assay

BMMs (5 × 10^4^ cells/well) were cultured in a 96-well plate (NUNC, Roskilde, Denmark) with *α*-MEM containing 10% FBS and 30 ng/mL of M-CSF in the presence of the respective saikosaponins at 1 and 10 *μ*M for 5 days. BMMs were also treated with saikosaponin A or D at the indicated concentrations for 5 days with replacement of fresh medium every second day. MDA-MB-231 cells (1 × 10^4^ cells/well) were seeded into 96-well plates with DMEM containing 10% FBS and then incubated in serum-free media with the indicated concentrations of saikosaponin A, saikosaponin D, and/or TGF-*β* (10 ng/mL) for 24 h. Cell viability was determined using the MTT assay as described previously [25] and expressed as percentage of control.

### 2.4. Osteoclast Formation Assay

BMMs (5 × 10^4^ cells/well) were cultured in a 96-well plate with the medium containing 10% FBS, M-CSF (30 ng/mL), RANKL (100 ng/mL), and saikosaponins at 5 *μ*M. In the other experiments, BMMs were treated with saikosaponin A or D at the indicated concentrations. Cultures were fed every 2 days with fresh medium for 5 days. The cells were stained for tartrate-resistant acid phosphatase (TRAP) using the Acid Phosphatase Leukocyte kit (Sigma-Aldrich). The total number of TRAP-positive multinucleated (≥3 nuclei) cells as osteoclasts per well was counted.

### 2.5. Gelatin Zymography and Cathepsin K Assay

BMMs (2 × 10^6^ cells/well) were seeded in a 6-well culture plate and incubated in medium containing 10% FBS, M-CSF (30 ng/mL), RANKL (100 ng/mL), and saikosaponin A or D at the indicated concentrations for 5 days. Cultures were fed every 2 days with fresh medium. The conditioned media were collected and then concentrated by centrifugation in a centricon centrifugal filter device for 30 min at 2,450 ×g at 4°C. The protein concentration in the conditioned media was determined with BCA protein assay reagents (Pierce, Rockford, IL). The concentrated culture media (35 *μ*g of proteins) were subjected to 8% SDS-polyacrylamide polyacrylamide gel containing 0.2% (w/v) gelatin, respectively. After electrophoresis, the gel was washed twice with 2.5% Triton X-100 for 1 h at room temperature and, subsequently, incubated for 24 h at 37°C in a reaction buffer containing 50 mM Tris-HCl (pH 7.5), 5 mM CaCl_2_, 200 mM NaCl, and 0.02% Brij-35. The gel was stained with 0.1% Coomassie brilliant blue R-250. Clear zones against the blue background indicated the presence of gelatinolytic activities.

Cathepsin K was detected using the Sensizyme cathepsin K activity assay kit (Sigma-Aldrich, MO) according to the manufacturer's instructions. The concentrated culture media were added to each well of a 96-well plate coated with cathepsin K antibody and incubated for 1 h at room temperature. After washing with PBS, the wells were incubated with the reaction mixture for 2 h. Absorbance was measured at 405 nm and the amount of cathepsin K in the conditioned medium was calculated as pg/mg protein with a standard curve.

### 2.6. Pit Formation Assay

BMMs (5 × 10^4^ cells) were seeded into Biocoat Osteologic MultiTest Slides (BD Biosciences, San Diego, CA), consisting of submicron synthetic mineralized calcium phosphate thin films coated with various culture vessels as described previously [25]. The cells were cultured in medium with 10% FBS, M-CSF (30 ng/mL), and RANKL (100 ng/mL) for 5 days and then treated with saikosaponin A or D at 3 and 5 *μ*M for an additional 11 days. Cultures were fed every 2 days with fresh medium. The cells were lysed with 5% sodium hypochlorite solution. The images of the resorbed pits were obtained under light microscopy (original magnification, ×100).

### 2.7. Cell Invasion Assay

The invasive ability of cells was investigated with a 24-well transwell chamber (Corning Costar, Cambridge, MA) containing polycarbonate membrane filter (6.5 mm diameter and 8 *μ*m pore size). The lower and upper surfaces of polycarbonate filter inserts were coated with 0.1% (w/v) gelatin and 1 mg/mL Matrigel (BD Biosciences, Palo Alto, CA) diluted with medium, respectively. Suspensions of MDA-MB-231 cell (5 × 10^4^ cells/0.1 mL) in serum-free medium with 0.1% BSA and the indicated concentrations of saikosaponin A or D were placed in the Matrigel-coated upper part. The lower chamber was filled with 600 *μ*L of medium containing 1% FBS and the indicated concentrations of saikosaponin A or D in the absence or presence of 10 ng/mL TGF-*β*. After 24 h of incubation, the cells were fixed with 70% methanol and stained with hematoxylin. Noninvaded cells on the upper surface of membrane were gently wiped off using a cotton swab, and invaded cells on the lower surface of membrane were mounted on slides. The stained cells were counted and photographed (original magnification, ×200) using a Zeiss Axio imager microscope (Carl Zeiss, Gottingen, Germany).

### 2.8. RNA Isolation and Quantitative Real-Time RT-PCR

MDA-MB-231 cells (5 × 10^5^ cells/dish) were seeded in 60 mm culture dishes and exposed to TGF-*β* (10 ng/mL) and saikosaponin A or D at indicated concentrations for 24 h. Total RNA was isolated from MDA-MB-231 cells using the RNeasy mini kit (Qiagen, Valencia, CA). RNA was converted to cDNA and then amplified using PrimeScript RT reagent kit (Takara Bio, Shiga, Japan). Quantitative real-time PCR analysis was conducted using 7300 Real-Time PCR System (Applied Biosystems, Foster City, CA, USA). The thermocycler parameters were 95°C for 30 sec, followed by 40 cycles of 95°C for 5 sec and 60°C for 31 sec. Glyceraldehyde-3-phosphate dehydrogenase (GAPDH) was used for normalization of mRNA analysis. The cycle threshold (Ct) was defined as 35 for the ΔCt calculation. Primer sequences are as follows: PTHrP, GACGACACACGCACTTGAAA (sense) and GGTTGCTTCCGGAAAGTTG (antisense), and GAPDH, CATGAGAAGTATGACAACAGCCT (sense) and AGTCCTTCCACGATACCAAAGT (antisense).

### 2.9. Statistical Analysis

Data are expressed as the mean ± standard error (SE) of three independent experiments. The statistical significance of the difference was analyzed by repeated measures of one-way analysis of variance followed by Student's *t*-test. Values of *P* < 0.05 were considered statistically significant.

## 3. Results

### 3.1. Effects of Saikosaponins on the Viability of BMMs

**Figure 2:**
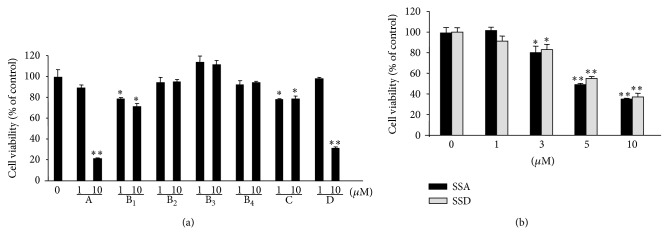
Cytotoxicity of saikosaponins on BMMs. (a) BMMs (5 × 10^4^ cells/well) were incubated in *α*-MEM with 10% FBS, M-CSF (30 ng/mL), and each saikosaponin (1 and 10 *μ*M) for 5 days. (b) BMMs were treated with saikosaponin A (SSA) or D (SSD) at the indicated concentrations for 5 days. Cell viability was determined by MTT assay. The data are expressed as the mean ± SE. ^*^ *P* < 0.05 and ^**^ *P* < 0.001 versus BMMs without saikosaponins.

The cytotoxicity in BMMs when exposed to the seven* B. falcatum-*derived saikosaponins was determined by the MTT assay. At 10 *μ*M concentrations, saikosaponins B_1_ and C had weak cytotoxicity (71–79% cell viability) and saikosaponins B_2_, B_3_, and B_4_ did not have any effect on cell viability (Figure 2(a)). Saikosaponins A and D markedly decreased cell viability when the cells were exposed to more than 3 *μ*M (Figure 2(b)) with IC_50_ values of 5.2 and 5.4 *μ*M, respectively.

### 3.2. Effects of Saikosaponins on Osteoclast Formation in RANKL-Treated BMMs

**Figure 3:**
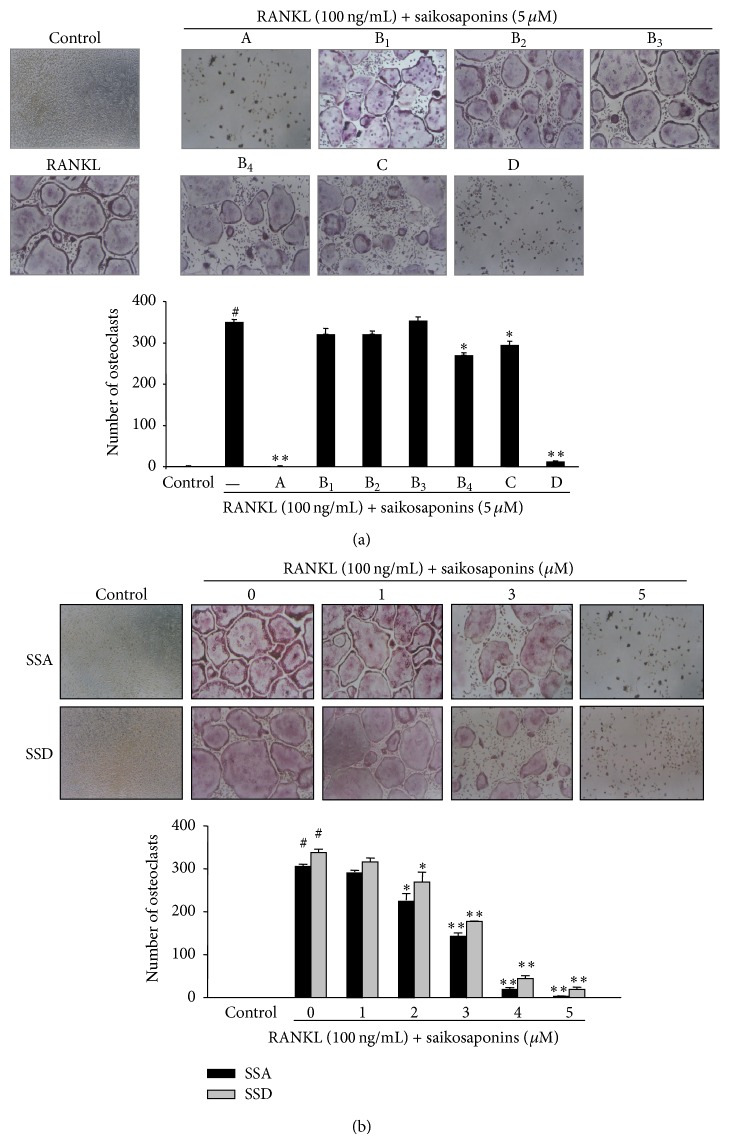
Effects of saikosaponins on RANKL-induced osteoclast formation in BMMs. (a) BMMs (5 × 10^4^ cells/well) were cultured in 10% FBS, *α*-MEM with M-CSF (30 ng/mL), RANKL (100 ng/mL), and each saikosaponin (5 *μ*M). (b) BMMs were treated with saikosaponin A (SSA) or D (SSD) at the indicated concentrations. Control cells were treated with M-CSF alone. Five days later, RANKL-treated BMMs were stained for TRAP. TRAP-positive multinucleated (>3 nuclei) cells were photographed (original magnification, 100x) and counted. The number of osteoclasts is expressed as the mean ± SE. ^**#**^ *P* < 0.0001 versus control and ^*^ *P* < 0.05 and ^**^ *P* < 0.001 versus RANKL-treated BMMs.

We next investigated the activity of the saikosaponins on RANKL-induced osteoclastogenesis. Treatment with saikosaponin A or D at 5 *μ*M almost completely inhibited osteoclast differentiation in BMMs treated with M-CSF and RANKL for 5 days. Saikosaponins B_4_ and C reduced osteoclast formation by 23 and 17%, respectively; however, saikosaponins B_1_ and B_2_ did not show any significant inhibition (Figure 3(a)). Furthermore, we confirmed that saikosaponins A and D reduced RANKL-induced osteoclast formation in a dose-dependent manner (Figure 3(b)). The IC_50_ values for saikosaponins A and D were 3.69 and 3.85 *μ*M, respectively.

### 3.3. Effects of Saikosaponins A and D on the Formation of Resorption Pits

**Figure 4:**
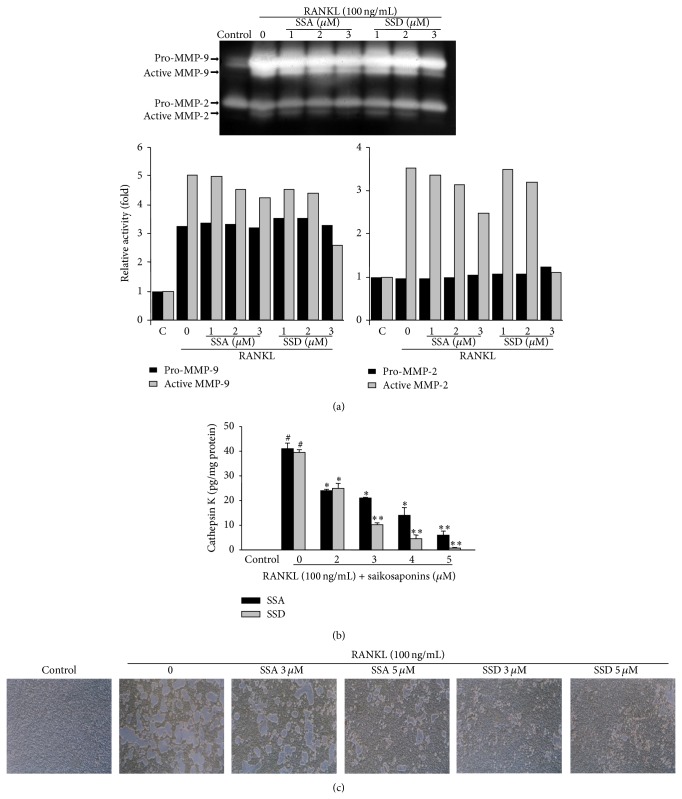
Effects of saikosaponins A and D on osteoclasts activity and the formation of resorption pits. BMMs (2 × 10^6^ cells/well) were seeded into a 6-well culture plate and incubated in a medium containing M-CSF (30 ng/mL), RANKL (100 ng/mL), and saikosaponin A or D for 5 days. Control cells were cultured in the presence of M-CSF alone. The culture medium was collected and concentrated. (a) The levels of MMP-2 and MMP-9 in the conditioned medium were determined by gelatin zymography. Relative activity was described as fold by densitometric analysis. (b) Cathepsin K level was detected with the Sensizym cathepsin K activity assay kit. (c) BMMs (5 × 10^4^ cells/well) were treated with M-CSF (30 ng/mL), RANKL (100 ng/mL), and the indicated concentrations of saikosaponin A (SSA) or D (SSD) on Biocoat Osteologic Multitest slides for 11 days after the induction of osteoclast formation. Control cells were treated with M-CSF alone. Cells were lysed and the pit areas were observed under phase-contrast microscopy. Data are expressed as the mean ± SE. ^#^ *P* < 0.05 versus control and ^*^ *P* < 0.05 and ^**^ *P* < 0.001 versus RANKL-treated BMMs.

Proteases produced by the activated osteoclasts were analyzed in the cultured medium of BMMs treated with RANKL in the presence of saikosaponins for 5 days. Gelatin zymography indicated that RANKL treatment noticeably increased the levels of pro- and active forms of matrix metalloproteinase (MMP)-2 and MMP-9; however, saikosaponin A or D treatment suppressed the RANKL-induced levels of pro- and active MMP-9 in a dose-dependent manner in the conditioned medium of BMMs. The RANKL-induced level of active MMP-2 was also reduced by these saikosaponins but that of pro-MMP-2 was not (Figure 4(a)). Moreover, we found that the significantly increased cathepsin K level in the conditioned medium of RANKL-treated BMMs was dose-dependently inhibited by treatment with saikosaponin A or D (Figure 4(b)). The inhibitory effect of saikosaponins A and D on the bone-resorptive activity of RANKL-induced osteoclasts was determined by the pit formation assay. BMMs were treated with M-CSF and RANKL for 5 days to induce osteoclast formation and then exposed to saikosaponin A or D in the presence of M-CSF and RANKL for 11 days. Treatment with saikosaponin A or D at 3 and 5 *μ*M substantially reduced the formation of resorption pits compared to the vehicle-treated slides (Figure 4(c)).

### 3.4. Effects of Saikosaponins A and D on the Viability, Invasion, and PTHrP mRNA Expression in MDA-MB-231 Human Metastatic Breast Cancer Cells

**Figure 5:**
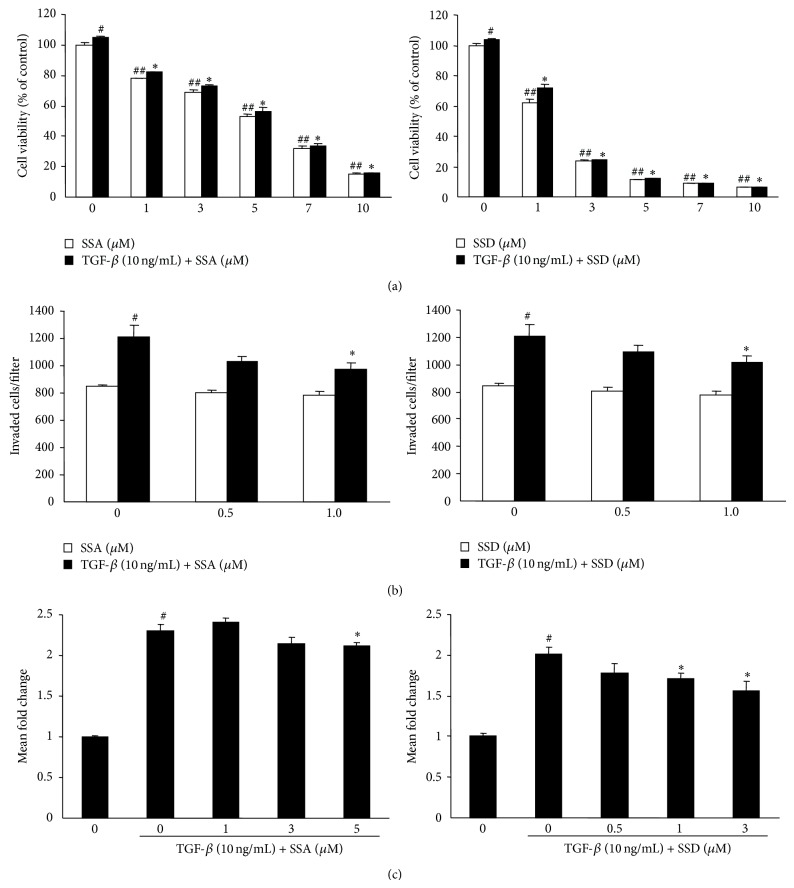
Effects of saikosaponins A and D on cell viability, invasion, and PTHrP mRNA expression in MDA-MB-231 metastatic human breast cancer cells. (a) MDA-MB-231 cells were treated with saikosaponin A, saikosaponin D, and/or TGF-*β* (10 ng/mL) for 24 h. Cell viability was determined by MTT assay. (b) Cell invasion assay was performed using a transwell chamber containing a polycarbonate membrane filter (6.5 mm diameter and 8 *μ*m pore size), coated with 0.1% (w/v) gelatin and Matrigel, as described in Materials and Methods. MDA-MB-231 cell suspensions with the indicated concentrations of saikosaponin A or D were added into the coated insert and the lower chamber was filled with 600 *μ*L of medium containing 1% FBS, saikosaponin A or D, and TGF-*β* (10 ng/mL). Transwell plates were incubated for 24 h. After hematoxylin staining, the membranes with invaded cells were mounted on slides. (c) PTHrP mRNA expression was determined by quantitative real-time RT-PCR in MDA-MB-231 cells treated with TGF-*β* (10 ng/mL) and saikosaponin A or D at indicated concentrations for 24 h. GAPDH was used for normalization of mRNA analysis. The gene expression levels are indicated as fold changes. Data are expressed as mean ± SE. ^#^ *P* < 0.05 and ^##^ *P* < 0.005 versus untreated cells and ^*^ *P* < 0.05 and ^**^ *P* < 0.005 versus TGF-*β*-treated cells.

TGF-*β* is very abundant growth factor in bone and promotes colonization, invasion, and osteolysis of bone metastases [26]. Saikosaponins A and D dose-dependently inhibited the viability of MDA-MB-231 cells (IC_50_ = 5.1 *μ*M for saikosaponin A, 2.3 *μ*M for saikosaponin D). TGF-*β* significantly increased cell viability but TGF-*β*-induced cell viability was decreased by 22%, 47%, and 85% by treatment with saikosaponin A at 1, 5, and 10 *μ*M and by 32% and 80% by treatment with saikosaponin D at 1 and 3 *μ*M, respectively (Figure 5(a)). Treatment with saikosaponin A or D at 1 *μ*M significantly reduced the number of invaded cells in TGF-*β*-stimulated cells (Figure 5(b)). In addition, TGF-*β* upregulated PTHrP mRNA expression but saikosaponins A and D significantly reduced TGF-*β*-induced PTHrP mRNA expression (Figure 5(c)).

## 4. Discussion

Bone morphogenesis and remodeling is a physiologically controlled process that involves the synthesis of bone matrix by osteoblasts and osteoclast-mediated bone resorption. Imbalances between osteoclast and osteoblast activities can arise from a wide variety of hormonal changes or perturbations in inflammatory and growth factors, resulting in skeletal abnormalities characterized by decreased (osteoporosis) or increased (osteopetrosis) bone mass. Increased osteoclast activity is seen in many osteopenic disorders such as postmenopausal osteoporosis, Paget's disease, lytic bone metastases in breast or prostate cancers, or increased bone resorption and crippling bone damage in arthritis [26]. The differentiation of osteoclast precursors into mature multinucleated osteoclasts is stimulated by M-CSF and RANKL [27]. RANKL, expressed in bone marrow stromal cells and osteoblasts, is a critical factor to form osteoclasts and binds its receptor RANK on osteoclast precursor cells. The RANKL-RANK binding triggers osteoclastogenesis by transducing a signal into the osteoclast precursors. Therefore, blockade of the RANKL-induced osteoclastogenesis is a promising strategy for reversing the onset and progression of osteoclast-mediated bone diseases. This study was designed to estimate the potential of saikosaponins as novel agents for preventing and treating osteoporosis and cancer-associated bone diseases. Of the seven saikosaponins, saikosaponins A and D had the strongest inhibition on osteoclast formation in RANKL-treated BMMs and substantially blocked the formation of osteoclast-mediated resorption pits on calcium phosphate-coated slides. These results suggest that saikosaponins A and D are potent antiosteoclastogenic and antibone resorptive agents.

Osteoclast-mediated bone resorption is generated by the removal of both mineral and organic constituents of the bone matrix. Osteoclasts produce various proteinases that efficiently degrade the matrix of bone. The degradation of organic matrix in bone is principally caused by MMPs and cathepsin K [28, 29]. MMPs are crucial for launching bone resorption by eradicating the collagenous layer from bone surface before starting demineralization [30]. Among several MMPs, MMP-9 is the most important proteinase involved in bone resorption as osteoclasts secrete this enzyme at a tremendously high level [29, 31]. Cathepsin K, a member of the papain cysteine protease superfamily, is highly expressed by mature osteoclasts [32]. The overexpressed cathepsin K stimulates bone resorption by degrading matrix proteins of bone, such as type I and type II collagen, osteopontin, and osteonectin, at low pH [32]. Recently, cathepsin K inhibitors as antiresorptive agents have been tested in clinical trials; however, this class of drug caused morphea [33, 34]. Our data indicated that saikosaponin A or D treatment reduced the levels of MMP-2, MMP-9, and cathepsin K secreted from RANKL-treated BMMs in a dose-dependent manner. These results suggest that saikosaponins A and D may inhibit bone resorption by reducing the secreted levels of MMP-2, MMP-9, and cathepsin K from mature osteoclasts derived from RANKL-treated BMMs.

Saikosaponin triterpenes generally constitute the main class of secondary metabolites in the genus* Bupleurum* amounting to up to 7% of the total dry weight in roots [13]. Saikosaponins can be divided into six types on the basis of the aglycones [19]. In this study, we isolated three types of saikosaponins: type I (saikosaponins A, D, and C) with an allyl oxide linkage in the 13, 28-position as the major saponins present in* Bupleurum* and maker compounds for quality control of closely related* Bupleurum* species, type II (saikosaponins B_1_ and B_2_) with a heteroannular diene structure, and type III (saikosaponins B_3_ and B_4_) with a 11-OCH_3_ [11]. Type I saikosaponins substantially inhibited osteoclast formation in RANKL-treated BMMs compared to other types of saikosaponins. In type I saikosaponins, saikosaponins A and D, with two carbohydrate chains that are directly attached to the hydroxyl groups in position 3, showed more potent inhibition on RANKL-induced osteoclastogenesis compared to saikosaponin C with three carbohydrate chains. These results suggest that the potent antibone resorptive activity of saikosaponins A and D is closely associated with both an allyl oxide linkage at the 13, 28-position and *β*-D-glucose-(1→3)-*β*-D-fucose at the 3-position; however, the stereochemistry of the hydroxyl group at C16 does not affect their activity.

In a vicious cycle of breast cancer bone metastasis, many growth factors and cytokines are released from bone matrix during osteoclastic bone resorption. These molecules cause severe bone loss by stimulating tumor growth in bone and production of tumor-derived osteolytic factors, inducing osteoblastic RANKL expression [35]. In particular, TGF-*β* increases tumor cell invasiveness and the production of several osteolytic factors, including PTHrP [36]. Our data indicated that saikosaponins A and D reduced cell viability and invasion and upregulated PTHrP mRNA expression in TGF-*β*-stimulated MDA-MB-231 metastatic human breast cancer cells.

## 5. Conclusion

Saikosaponins A and D inhibited RANKL-mediated osteoclast formation in BMMs and osteoclast-induced bone resorption by reducing the levels of MMP-2, MMP-9, and cathepsin K at noncytotoxic concentration. In addition, saikosaponins A and D inhibited TGF-*β*-induced cell invasion and PTHrP mRNA expression in MDA-MB-231 metastatic human breast cancer cells. Therefore, saikosaponins A and D could be potential therapeutic agents for the inhibition of osteoclast-mediated bone diseases, including osteoporosis and cancer cell-related bone loss.
